# Efficacy and safety of Qingre-Chushi therapies in active ulcerative colitis: A network meta-analysis

**DOI:** 10.1371/journal.pone.0257599

**Published:** 2021-09-20

**Authors:** Ling Zhang, Yun-bo Wu, Yun-kai Dai, Qi Liu, Yu-jie Ren, Shi-jie Xu, Huai-geng Pan, Wei-jing Chen, Ru-liu Li, Ling Hu

**Affiliations:** 1 Institute of Gastroenterology, Science and Technology Innovation Center, Guangzhou University of Chinese Medicine, Guangzhou, Guangdong Province, China; 2 Science and Technology Innovation Center, Guangzhou University of Chinese Medicine, Guangzhou, Guangdong Province, China; Bhagwan Mahvir College of Pharmacy, INDIA

## Abstract

**Background:**

Ulcerative colitis (UC) is a chronic inflammatory disease with an increasing incidence in the world. Qingre-Chushi therapies (QC) can alleviate clinical symptoms. Therefore, a network meta-analysis was conducted to systematically evaluate the efficacy and safety of QC in the treatment of active UC patients.

**Methods:**

7 databases were screened and relevant randomized controlled trials were selected. The tools of Cochrane Handbook and the GRADE system were conducted to assess the quality of outcomes. Pooled risk ratio or standard mean difference was calculated with 95% credible interval for outcomes measurement using the random-effects model. The surface under the cumulative ranking curve (SUCRA) was performed to rank the treatments. The larger SUCRA scores, the more effective interventions.

**Results:**

A total of 3560 articles were identified and 21 studies including 1829 participants were included for further analysis. Totally, 9 therapies regimens were compared: oral mesalazine, mesalazine enema, mesalazine suppository, oral mesalazine + mesalazine enema, oral QC, oral QC + oral mesalazine, QC enema, oral QC + QC enema, and oral mesalazine + QC enema. Based on the SUCRA plot, oral QC + oral mesalazine was the best treatment in inducing clinical response; oral QC + QC enema had the best efficacy in the improvement of Mayo scores and alleviating abdominal pain; oral mesalazine + mesalazine enema was the optimal therapy in the endoscopic improvement and reducing diarrhea; QC enema + oral mesalazine was the best option in preventing bloody stool.

**Conclusion:**

This study confirmed the efficacy and safety of QC in treating active UC and suggested that the combination of oral medications with topical can achieve more benefits.

## Introduction

Ulcerative colitis (UC) is a chronic inflammatory disease affecting the colon [1]. It is characterized by diffused mucosal inflammation, starting from the rectum and extending to proximal segments of the colon. The incidence and prevalence increase over time worldwide: the highest annual incidence of UC was 24.3 per 100,000 person-years in Europe, 6.3 per 100,000 person-years in Asia and the Middle East, and 19.2 per 100,000 person-years in North America [2]. Diarrhea and bloody stool are the most common clinical presentations of UC. However, the exact pathogenesis of UC is unclear and risk factors can be concluded as follow: genetic predisposition, infections, environmental factors, drugs, and appendectomy [3–8]. Due to the unclear mechanism and pathogenesis, the main goal of therapy is to induce and maintain clinical remission (Mayo scores≤2) with the long-term goals of preventing disability, colectomy, and colorectal cancer [9].

Mesalazine is the first-line medication in the treatment of mild to moderate active UC according to the guidelines from the European Crohn’s and Colitis Organization [10], and it can be administrated as a suppository, foam, enema, and oral formulation based on the extension of disease. Patients with moderate to severe UC should be treated with systemic corticosteroids, thiopurines, anti-TNF drugs, calcineurin Inhibitors, anti-adhesion molecule inhibitors, or Janus Kinase Inhibitor [1, 11]. But the application of these conventional drugs often accompanies with adverse events which affect patient’s quality of life, and the long-term efficacy is unsatisfactory [12, 13]. Therefore, it’s necessary to look for another alternative complementary therapy.

Traditional Chinese medicine (TCM) has been used to treat symptoms associated with UC for literally thousands of years. In recent years, several studies have suggested the therapeutic effects of Chinese herbal medicine (CHM) formulae in the treatment of UC [14–16]. Meanwhile, some systematic reviews and pairwise meta-analysis have also concluded the effects of some CHM formulae in treating UC patients [17–19]. In TCM theory, syndrome differentiation is the core of diseases, playing an important role in the pathogenesis and curation. A retrospective review confirmed that damp-heat accumulating is the most common syndrome (6153/10749) in patients with UC [20]. Clinically, clear heat and eliminate dampness (TCM jargon: Qingre-Chushi therapies, QC) therapies are one of the most commonly used treatments for UC patients [21, 22]. However, the efficacy and safety of QC have not been systemically concluded yet. Based on these, a network meta-analysis (NMA) was conducted to explore the therapeutic effects and safety of QC.

## Methods

This study was performed in conformity to the Cochrane Handbook for the Systematic Review of Interventions and the Preferred Reporting Items for Systematic Review and Meta-Analyses (PRISMA) [23]. This protocol has been registered on PROSPERO CRD42020204540 (https://www.crd.york.ac.uk/prospero/#searchadvanced). The PRISMA checklist was reported in S1 Table. No further ethical approval is required since all eligible studies were approved by local institutional review boards and ethical committees.

### Information sources and search strategy

In total, the following databases were screened from their inception to September 15, 2020: PubMed, Springer, Embase, Cochrane Library, China National Knowledge Infrastructure (CNKI), Chinese Biomedical Literature database (CBM), and WanFang. Language restriction was not applied. The detailed search strategy was summarized in S1 File.

### Study selection and outcome assessment

Relevant randomized controlled trials (RCTs) meeting the following inclusion criteria were enrolled: (1) Patients: adults aged 18–70 with a definite diagnosis of active UC (Mayo clinical score>2). (2) Intervention: any prescriptions of QC therapies with a minimum duration of 4 weeks. The QC therapies refer to any prescriptions mainly composed of Chinese herbs that can clear heat or eliminate dampness. (3) Comparator: conventional medications of UC. The QC therapies were included in the treatment groups, and the formulations were not limited. Conventional therapies such as mesalazine, sulfasalazine, or steroid, were compared as the control group, and they can be administrated as an oral pill, enema, suppository, or combination. (4) Outcome: clinical response; improvement of Mayo score; endoscopic improvement; TCM clinical syndrome integral. The specific definition of outcomes assessment was shown in S2 File. Exclusion criteria were as follows: pregnant women; patients with steroid-dependent or acute severe UC; patients with severe complications; studies with Jadad scores< 3.

### Data extraction and quality evaluation

Two investigators independently conducted data extraction and crosschecked. Judgments were independently performed by two investigators and disagreements were remedied after discussing with a third investigator. Data extraction included the following items:(1) general information: topic, authors, publication date, etc. (2) clinical characteristic of trials: gender, age, intervention, etc. (3) methodological heterogeneity of trials: randomization, double-blind, withdrawal or dropout, etc. (4) outcomes evaluation index: clinical response, etc. The methodological quality of each included study was estimated based on the Cochrane Collaboration Recommendations assessment tool [24]. In addition, the strength of evidence will be evaluated by the Grading of Recommendations Assessment, Development, and Evaluation (GRADE) approach [25].

### Statistical analysis

A network meta-analysis with the Bayesian framework was conducted to provide more precise evidence about the comparative efficacy and safety of different treatments [26]. We used WinBUGS software (version 1.4.3), based on the Bayesian framework and the Markov chain Monte Carlo method, to evaluate and process research data a priori. Stata16.0 and Review management 5.3 software were used to draw network diagrams and compare multiple interventions directly or indirectly. The node-splitting analysis was performed to test the consistency of the model. Were the *p*-value of the node-splitting analysis greater than 0.05, a consistency model would be chosen [27]. Otherwise, an inconsistency model was selected. The inconsistency index statistic (*I*^*2*^) and the *p*-value were used for evaluating heterogeneity and the random-effect model was selected accordingly [28]. The effects size for dichotomous variables such as clinical response and adverse effects will be calculated by risk ratio (RR) with 95% credible interval (CI). For continuous variables such as Mayo scores, endoscopic improvement, and TCM integral scores, the standard mean difference (SMD) with 95%CI was conducted. A funnel plot was applied to evaluate the existence of publication bias. The surface under the cumulative ranking curve (SUCRA) was calculated to rank the probability of interventions [29]. The larger SUCRA scores, the more effective interventions.

## Results

### Study selection

**Fig 1:**
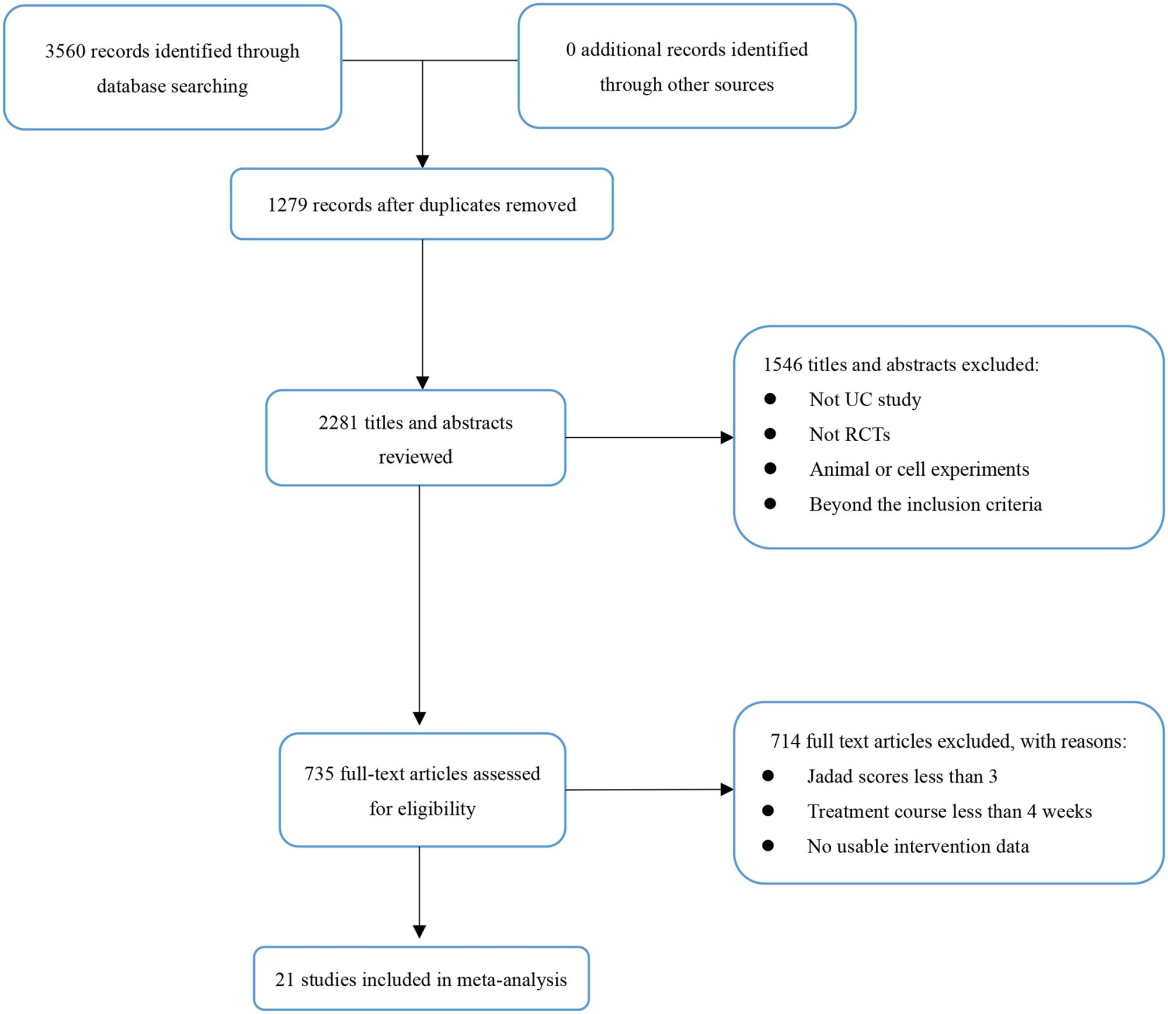
Flowchart of the process for literature retrieval. UC: Ulcerative colitis; RCTs: Randomized controlled trials.

A total of 3560 articles were identified from 7 databases and 21 RCTs including 1829 participants were included for further analysis [30–50]. The flowchart of database searching was summarized in Fig 1. The baseline characteristic of the included studies was concluded in Table 1. The datasets presented in this study and the usage of herbs can be founded in S2 Table.

**Table 1:** Characteristics of the studies included.

Study ID	Severity of UC	Endoscopic diagnostics (Montreal classification)	TCM criteria	Sample Size		Age (years)	Course of disease (years)	Duration (weeks)	Intervention		Follow-up (weeks)
				EG (M/F)	CG (M/F)				EG	CG	
**Fan et.al 2020**	S1:36 S2:29	E1:21 E2:35 E3:9	large intestinal dampness-heat	33 (18/15)	32 (18/14)	E:39 C:38	E:0.25~15 C:0.25–14	6	QC, qd, po.	E1: Mesalazine,0.5g/tid, sg E2, E3:Mesalazine,1g/qid, po	N/A
**Shan 2020**	S1/S2	E1/E2	large intestinal dampness-heat	30 (19/11)	30 (17/13)	E:39.51±3.01 C:40.11±3.53	E:3.66±1.34 C:3.21±1.36	4	Mesalazine,1g/tid, po. + QC 200ml/qd, pr.	Mesalazine,1g/tid, po +Mesalazine,1.0g/pr.	12
**Wang 2020**	S1:27 S2:34	E1:23 E2:38	spleen deficiency With dampness-heat	31 (17/14)	30 (16/14)	E:45.97±11.60 C:45.19±10.27	E:2.875±4.125 C:3.75±4.917	8	QC, 150ml/tid, po	Mesalazine, 1g/tid, po	12
**Xie et.al 2020**	S1:32 S2:34	E2:66	spleen deficiency With dampness-heat	33 (14/19	33 (16/17)	E:38.3±12.32 C:36.8±11.84	E:3.24±1.37 C:3.35±1.53	13	QC,100ml/bid, po. +QC, 100ml/q3w, pr	Mesalazine,1g/qid, po	N/A
**Zhong et.al 2020**	S1/S2	N/A	spleen deficiency With dampness-heat	15 (8/7)	C1: 15 (9/6) C2: 15 (9/6)"	E1:41.60±9.86 E2:44.60±12.21 C:39.67 ±12.32	N/A	8	QC, 2g/tid, po +Mesalazine, 1g/tid	C1: QC, 2g/tid, po C2: Mesalazine, 1g/tid	N/A
**Ding 2019**	S1:42 S2:38	E1:26 E2:34 E3:20	spreading dampness heat pattern	40 (18/22)	40 (21/19)	E:40.55±7.12 C:42.95±5.99	E:7.1±1.44 C:6.8±1.21	8	Mesalazine,1g/qid, po +QC, 100ml/bid, po.	Mesalazine,1g/qid, po	24
**Du 2019**	S1:25 S2:35	E1:60	large intestinal dampness-heat	30 (14/16)	30 (13/17)	E:37.0±10.18 C:35.7±10.63	E:2.19±1.14 C: 2.08±0.96	9	QC,50ml/qn, pr	Mesalazine,1g/qn, sg	N/A
**Jia 2019**	S1:26 S2:34	E1:20 E2:40	large intestinal dampness-heat	30 (14/16)	30 (12/18)	18~65	N/A	8	QC,100ml/qn, pr	Mesalazine,4g/qn, pr	8
**Wang 2019**	S1/S2	N/A	spleen deficiency With dampness-heat	31 (15/16)	30 (13/17)	E:46.16±9.73 C:44.60±9.74	N/A	8	QC, 150ml/tid, po.	Mesalazine, 1.5g/tid, po	N/A
**Wu 2019**	S2:30 S3:32	N/A	large intestinal dampness-heat	30 (14/16)	30 (11/19)	E:43.2±12.74 C:41.1±12.16	E:0.86±0.59 C:0.88±0.46	8	Mesalazine,1g/tid, po+QC,150ml/qd, pr	Mesalazine,1g/tid, po	12
**Zhang 2019**	S1:24 S2:37	E1:61	Dampness-heat syndrome	31 (13/18)	30 (14/16)	E:34.42±10.60 C:33.13±10.71	E:2.58±1.16 C:2.40±1.15	8	QC, 50ml/qn, pr	Mesalazine, 1g/qn, sg	N/A
**Feng 2018**	S1:18 S2:52	E1:19 E2:41 E3:10	large intestinal dampness-heat	35 (26/9)	35 (23/12)	E:42.4±11.65 C:41.9±13.25	E:3.0±2.06 C:3.1±1.92	12	QC,100ml/qd, po. +QC,100ml/q3w, pr	Mesalazine,0.5g/tid, po	N/A
**Yao 2018**	S1:25 S2:35	N/A	damp-heat accumulation interior pattern	30(12/18)	30(15/15)	E:39.48±5.65 C:40.67±3.21	E:6.18±1.27 C:6.68±0.34	8	QC, 50ml/qn, pr	Mesalazine, 1g/qn, sg	N/A
**Zhang et.al 2018**	S1:28 S2:37	N/A	large intestinal dampness-heat	33 (19/14)	32 (17/15)	E:42.96±9.56 C:41.2±10.12	E:4.7±5.52 C:4.3±5.99	8	QC,100ml/bid, po+QC,100ml, pr	Mesalazine,1g/qid, po	N/A
**Dai et.al 2017**	S1:73 S2:47	N/A	spleen deficiency and dampness-heat syndrome	60 (36/24)	60 (34/26)	E:38.2±11.7 C:40.5±12.3	E:4.55±3.24 C:4.23±3.55	8	QC,200ml/bid, po	Mesalazine,1.0g, qid, po.	N/A
**Qin 2017**	S1:16 S2:41 S3:3	E1:15 E2:37 E3:8	large intestinal dampness-heat	30 (17/13)	30 (17/13)	E:43.87±15.62 C:47.10±13.93	E:3.62±4.26 C:6.27±7.36	4	QC, 150ml/bid, po	Mesalazine, 1g/qid, po	N/A
**Bao 2015**	S1/S2	N/A	large intestinal dampness-heat	34 (24/10)	34 (20/14)	E:45.13±8.27 C:47.58±8.44	E:2.84±0.22 C:3.06±0.21	4	QC,250ml/bid, po	Mesalazine,0.5g/bid, po	N/A
**Yang et.al 2014**	S1/S2	N/A	N/A	32 (14/18)	32 (16/16)	E:38 C:40	E:3.67 C:4	8	QC,6g/qd, pr	Mesalazine, 1g/tid, po.	N/A
**Gong 2012**	S2/S3	N/A	damp-heat accumulation interior pattern	234 (123/111)	80 (38/42)	E:43.63 ± 12.01 C:44.51 ± 12.02	E: 2.99 ± 3.56 C: 2.39 ± 3.89	8	QC, 1.6g/tid, po	Mesalazine, 1g/qid, po	N/A
**Liu 2011**	S1:22 S2:33 S3:5	E1:11 E2:37	large intestinal dampness-heat	30 (16/14)	31 (17/14)	E:38.67±10.16 C:38.80±10.29	E:5.97±2.98 C:6.13±3.15	6	QC, 150ml/qd, po	Mesalazine, 1g/qid, po	N/A
**Tong et.al 2011**	S1:75 S2:66 S3:19	N/A	damp-heat accumulation interior pattern	120 (59/61)	40 (23/17)	E:42.88 ±11.77 C:42.70 ±10.42	N/A	8	QC,1.6g/tid, po.	Mesalazine, 1g/qid, po.	N/A

**Annotations:** UC = ulcerative colitis; EG = experiment group; CG = control group; N/A = not applicable; QC = Qingre-Chushi therapy; M = male; F = female;

### Risk of bias evaluation

**Fig 2:**
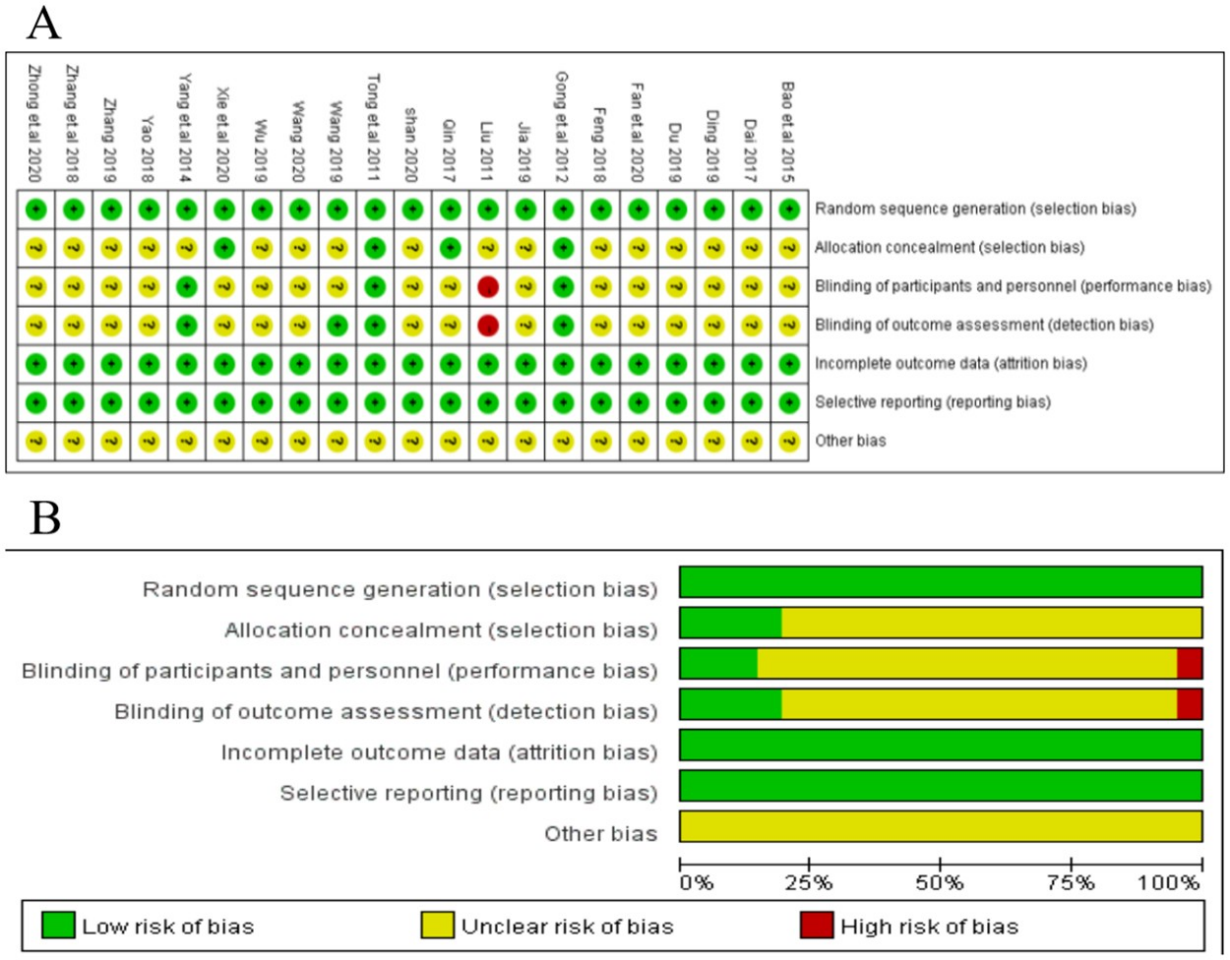
Risk of bias evaluation. (a) Risk of bias graph for each study included; (b) Risk of bias graph for all studies included.

The quality of the included RCTs was evaluated by the Cochrane Risk of Bias Assessment Tool, which includes the following factors:

1. selective bias (Random sequence generation and Allocation concealment): All the studies described the specific explanation of the generation of random sequences such as random number tables or a random number generated by computer. Therefore, all trials were assessed as “low risk”. For allocation concealment, only 4 trials reported detailed information and were considered as “low risk” while the rest 17 studies were assessed “unclear risk” because of insufficient information.
2. Performance bias (Blinding of participants and personnel) and detection bias (Blinding of outcome assessment): For performance bias, 3 studies provided information of blinding such as placebo-controlled and was evaluated as “low risk” while 1 trial was lack of blind method thus was estimated as “high risk”. The remaining 17 trials failed to report enough information, thus were assessed as “unclear risk”. For detection bias, 4 studies describe adequate information about blinding thus were considered as “low risk” while 1 study was open-label and was “high risk”. The rest of the 16 studies were estimated as “unclear risk” due to the lack of information.
3. Attrition bias (Incomplete outcome data): In total, all studies reported complete data or reported withdrawal or dropouts. Therefore, all trials were considered as “low risk”.
4. Reporting bias (Selective reporting): All studies were estimated as “low risk” in this item because of the acquired complete implementation scheme.
5. Other bias: Due to the lack of information in this item, all studies were considered as “unclear risk”. The overall quality assessment was shown in Fig 2.

### Network evidence

**Fig 3:**
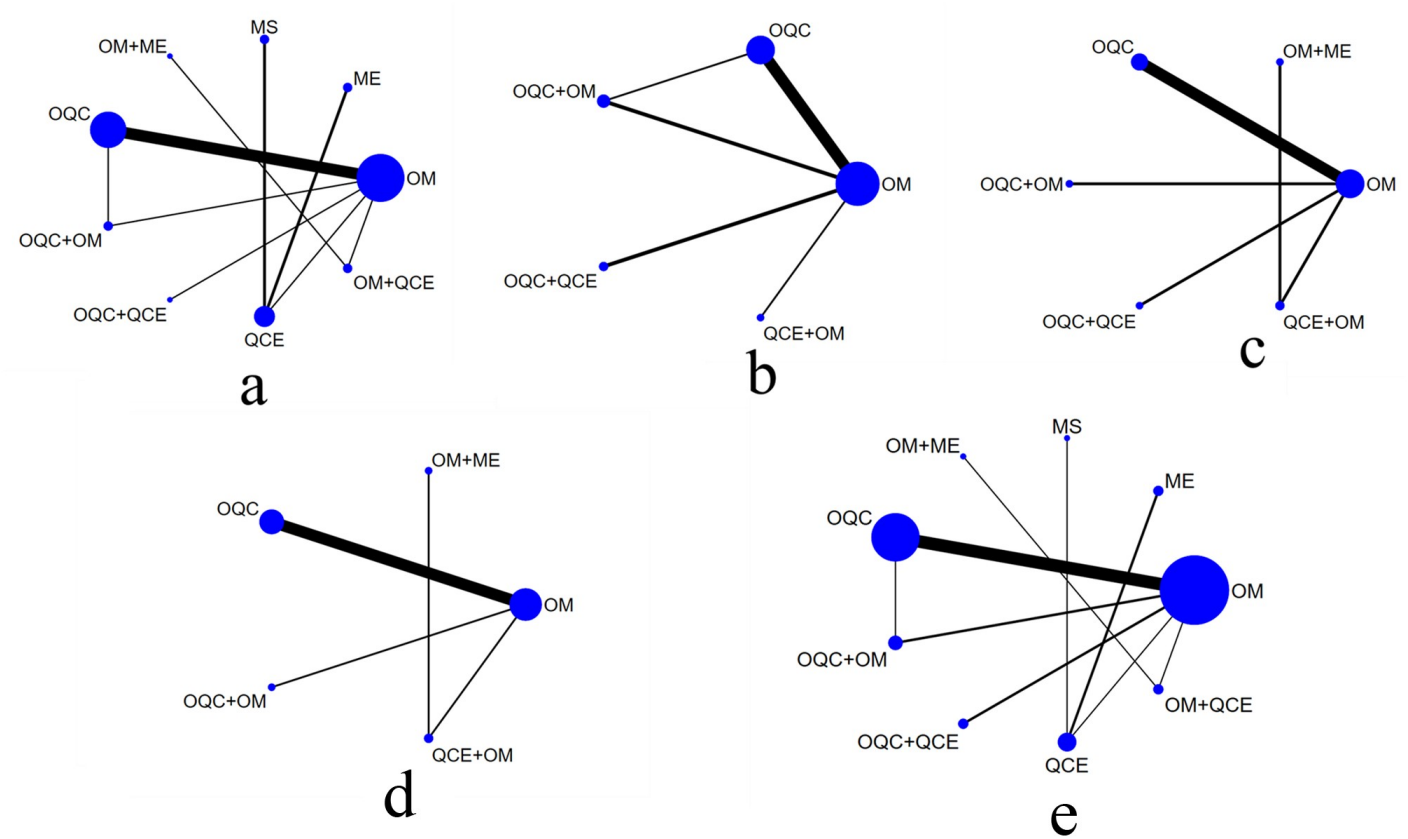
Network evidence of all endpoints. (a) Clinical response; (b) Improvement of Mayo scores; (c) Endoscopic improvement; (d) TCM syndrome integral; (e) Adverse effects. OM: Oral mesalazine; OQC: Oral Qingre-Chushi therapies; QCE: Qingre-Chushi therapies enema; ME: Mesalazine enema; MS: mesalazine suppository.

Totally, 9 therapies regimens were compared: oral mesalazine (OM), mesalazine enema (ME), mesalazine suppository (MS), oral mesalazine combined with mesalazine enema (OM+ME), oral QC (OQC), oral QC combined with oral mesalazine (OQC+OM), QC enema (QCE), oral QC combined with QC enema (OQC+QCE), and oral mesalazine combined with QC enema (OM+QCE).. The network graphs with different outcomes were displayed in Fig 3.

### Primary outcomes

#### Clinical response

**Fig 4:**
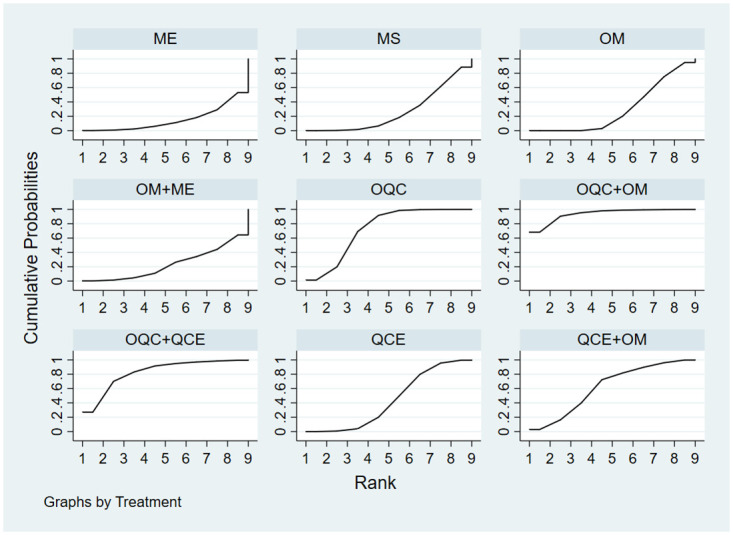
Surface under the cumulative raking curve of clinical response.

**Fig 5:**
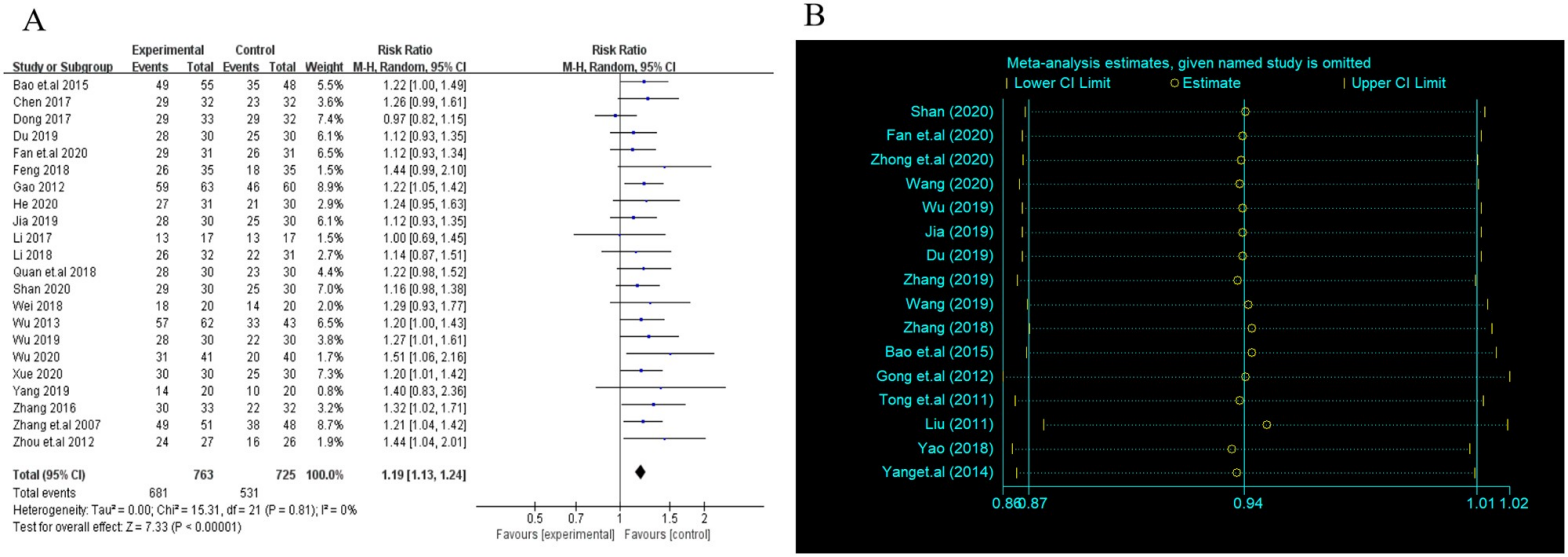
Heterogeneity analysis and sensitivity analysis. (A) Heterogeneity analysis of clinical response; (B) Sensitivity analysis of clinical response. CI: Confidence interval; OR: Odd ratio.

**Fig 6:**
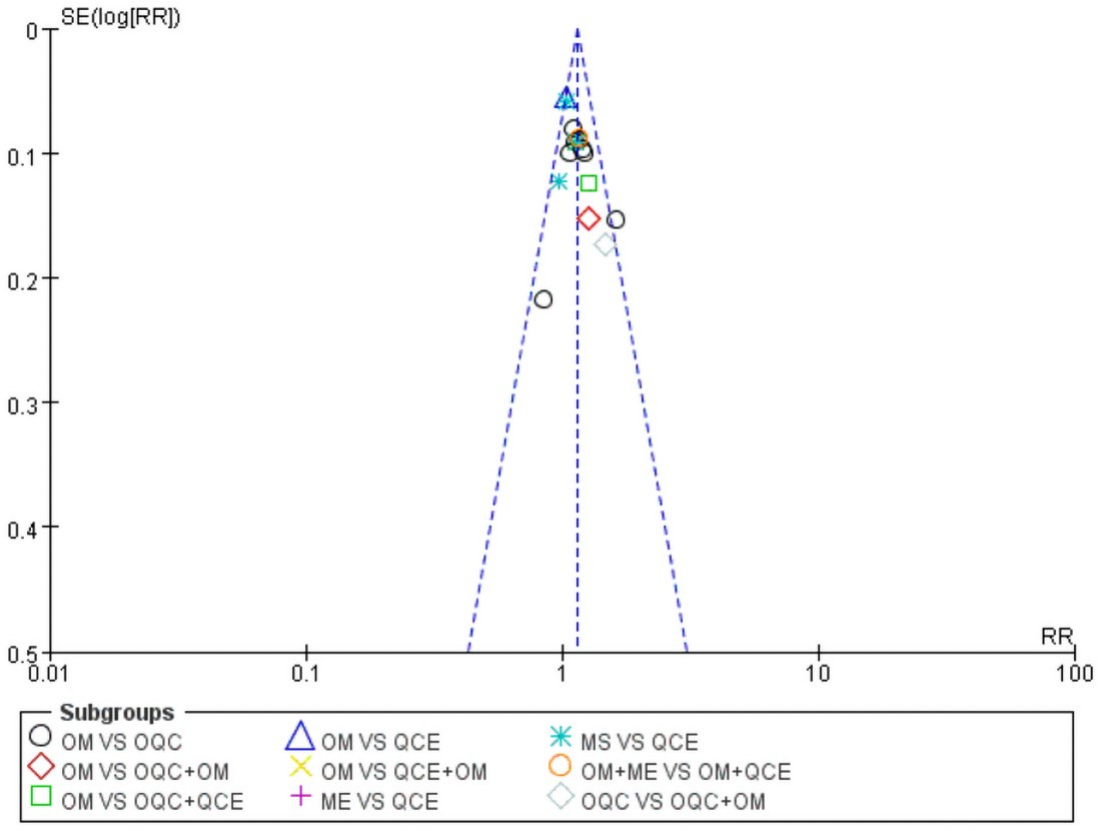
Funnel plot of clinical response. OM: Oral mesalazine; OQC: Oral Qingre-Chushi therapies; QCE: Qingre-Chushi therapies enema; ME: Mesalazine enema.

There were 16 trials reporting clinical response. As shown in Table 2, OQC+OM was superior to QCE, OM, ME, MS and OM+ME. Besides, OQC is better than OM. The SCURA plot in Fig 4 indicated that OQC+OM ranked first, followed by OQC+QCE and OQC. Besides, heterogeneity analysis (Fig 5A) showed good homogeneity (*I*^*2*^ = 0.0%, *P* = 1), and sensitivity analysis (Fig 5B) indicated strong stability. Meanwhile, the symmetry funnel plot was observed in Fig 6.

**Table 2:** Risk ratio (RR) or standard mean difference (SMD) with 95% confidence interval (CI) of different outcomes.

**Clinical response**								
**OQC+OM**								
1.10 (0.78,1.56)	**OQC+QCE**							
1.20 (0.93,1.55)	1.09 (0.85,1.41)	**OQC**						
1.25 (0.92,1.70)	1.13 (0.84,1.53)	1.04 (0.86,1.26)	**QCE+OM**					
**1.35 (1.03,1.77)**	1.22 (0.94,1.60)	1.12 (0.98,1.28)	1.08 (0.87,1.33)	**QCE**				
**1.39 (1.08,1.79)**	1.26 (0.99,1.61)	**1.16 (1.08,1.24)**	1.12 (0.93,1.34)	1.03 (0.93,1.15)	**OM**			
**1.41 (1.06,1.88)**	1.28 (0.97,1.69)	1.17 (1.00,1.37)	1.13 (0.90,1.42)	1.04 (0.95,1.14)	1.01 (0.88,1.16)	**MS**		
**1.45 (1.02,2.06)**	1.32 (0.93,1.86)	1.20 (0.93,1.56)	1.16 (0.98,1.38)	1.07 (0.82,1.41)	1.04 (0.81,1.34)	1.03 (0.77,1.37)	**OM+ME**	
**1.50 (1.08,2.08)**	1.37 (0.99,1.88)	1.25 (1.00,1.56)	1.20 (0.91,1.59)	1.12 (0.93,1.34)	1.08 (0.87,1.33)	1.07 (0.87,1.31)	1.04 (0.75,1.44)	**ME**
**Mayo scores**								
**OQC+QCE**								
1.42 (0.51,3.95)	**QCE+OM**							
1.73 (0.80,3.71)	1.22 (0.46,3.21)	**OQC+OM**						
**2.64 (1.35,5.19)**	1.87 (0.76,4.58)	1.53 (0.88,2.67)	**OQC**					
**3.21 (1.77,5.81)**	2.27 (0.98,5.24)	**1.86 (1.14,3.01)**	1.21 (0.88,1.67)	**OM**				
**Endoscopic improvement**								
**OM+ME**								
**6.65 (1.34,32.88)**	**OQC+QCE**							
**7.05 (2.47,20.14)**	1.06 (0.32,3.54)	**QCE+OM**						
**10.68 (2.19,52.07)**	1.61 (0.41,6.29)	1.51 (0.46,4.96)	**OQC+OM**					
**15.41 (3.85,61.68)**	2.32 (0.75,7.18)	2.18 (0.88,5.41)	1.44 (0.48,4.38)	**OQC**				
**16.22 (4.58,57.50)**	2.44 (0.92,6.48)	**2.30 (1.13,4.67)**	1.52 (0.59,3.94)	1.05 (0.60,1.86)	**OM**			
**Traditional Chinese medicine syndrome integral**								
**Abdominal pain**								
**OQC+QCE**								
1.08 (0.52,2.25)	**QCE+OM**							
1.14 (0.47,2.77)	1.05 (0.64,1.75)	**OM+ME**						
**1.79 (1.03,3.10)**	1.66 (0.93,2.98)	1.57 (0.73,3.41)	**OQC**					
**2.85 (1.72,4.70)**	**2.64 (1.54,4.52)**	**2.50 (1.20,5.24)**	**1.59 (1.26,2.00)**	**OM**				
**Diarrhea**								
**OM+ME**								
1.39 (0.65,2.98)	**OQC**							
1.48 (0.89,2.47)	1.07 (0.60,1.88)	**QCE+OM**						
1.75 (0.73,4.18)	1.26 (0.74,2.14)	1.18 (0.59,2.39)	**OQC+QCE**					
**2.81 (1.35,5.82)**	**2.02 (1.60,2.55)**	**1.90 (1.13,3.19)**	1.60 (1.00,2.58)	**OM**				
**Bloody stool**								
**QCE+OM**								
1.03 (0.62,1.71)	**OM+ME**							
1.17 (0.66,2.08)	1.14 (0.53,2.44)	**OQC**						
1.38 (0.68,2.80)	1.34 (0.56,3.19)	1.18 (0.69,1.99)	**OQC+QCE**					
**2.09 (1.23,3.52)**	2.02 (0.97,4.18)	**1.78 (1.41,2.24)**	1.51 (0.94,2.42)	**OM**				
**Adverse effects**								
**QCE**								
0.48 (0.05,4.54)	**OQC+QCE**							
0.45 (0.06,3.59)	0.93 (0.12,7.38)	**OQC+OM**						
0.35 (0.06,2.12)	0.72 (0.12,4.26)	0.77 (0.15,3.83)	**OQC**					
0.29 (0.00,91.97)	0.59 (0.00,189.46)	0.63 (0.00,191.21)	0.82 (0.00,226.88)	**OM+ME**				
0.29 (0.00,19.72)	0.59 (0.01,40.59)	0.63 (0.01,40.04)	0.82 (0.01,45.87)	1.00 (0.02,50.68)	**QCE+OM**			
0.29 (0.06,1.40)	0.59 (0.12,2.86)	0.63 (0.17,2.41)	0.82 (0.35,1.96)	1.00 (0.00,257.66)	1.00 (0.02,50.68)	**OM**		
0.20 (0.01,4.20)	0.41 (0.01,18.07)	0.44 (0.01,17.64)	0.58 (0.02,19.88)	0.70 (0.00,478.50)	0.70 (0.00,128.80)	0.70 (0.02,21.68)	**ME**	
0.17 (0.02,1.39)	0.34 (0.02,7.50)	0.37 (0.02,7.16)	0.48 (0.03,7.78)	0.58 (0.00,272.62)	0.58 (0.01,66.23)	0.58 (0.04,8.24)	0.83 (0.02,33.93)	**MS**

**Annotation**: OM: Oral mesalazine; OQC: Oral Qingre-Chushi therapies; QCE: Qingre-Chushi therapies enema; ME: Mesalazine enema; MS: Mesalazine suppository.

#### Mayo scores

**Fig 7:**
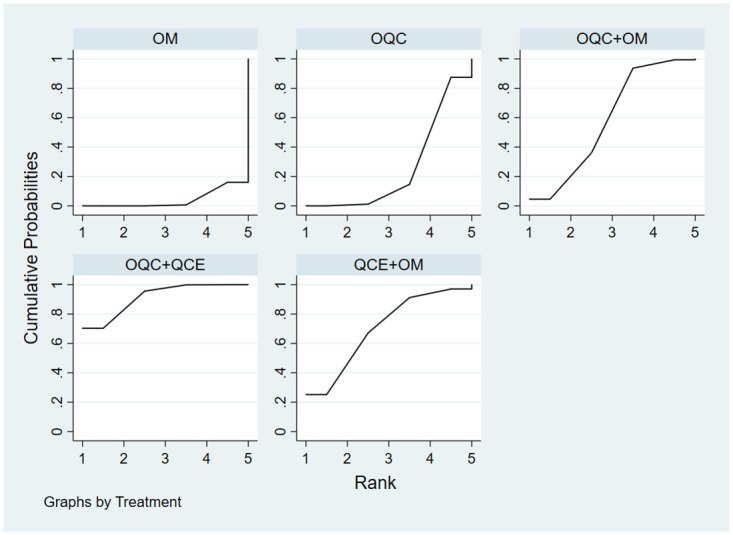
Surface under the cumulative raking curve of Mayo scores.

The improvement of Mayo scores was reported in 11 RCTs and involved 5 therapies. Compared with OM (Table 2), OQC+QCE (SMD = 3.21, 95%CI:1.77, 5.81) and OQC+OM (SMD = 1.86, 95%CI:1.14, 3.01) had better efficacy in improving Mayo scores. Additionally, OQC+QCE was superior to OQC (SMD = 2.64, 95%CI = 1.35, 5.19). Based on the SCURA plot (Fig 7), OQC+QCE was the best intervention, while QCE+OM, OQC+OM ranked 2^nd^ and 3^rd^.

### Secondary outcomes

#### Endoscopic improvement

**Fig 8:**
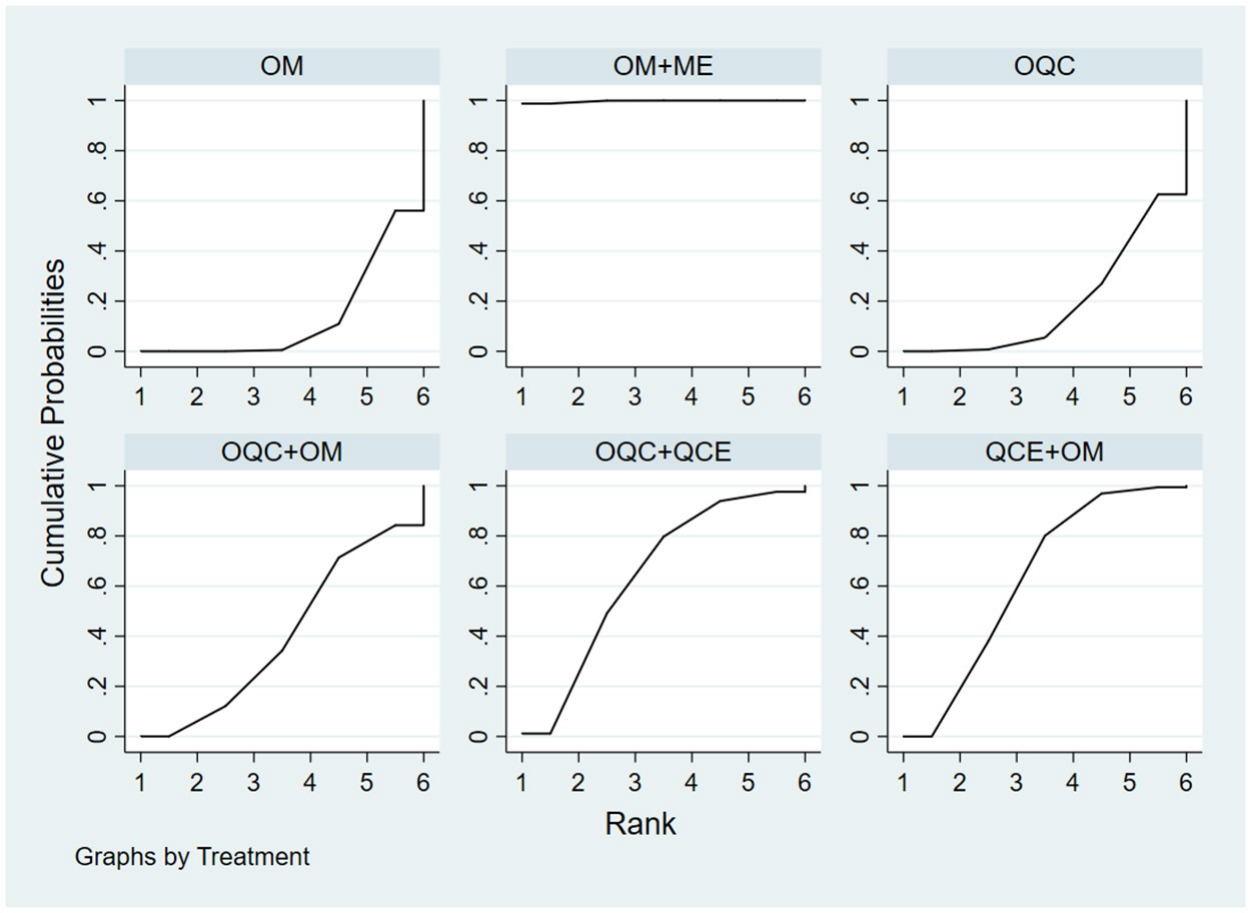
Surface under the cumulative raking curve of endoscopic improvement.

In total, 8 trials that involved 6 treatments reported endoscopic improvement. The result in Table 2 showed that OM+ME is the optimal therapy among all other therapies. Besides, QCE+OM is more favorable than OM (RR = 2.30, 95%CI = 1.13, 4.67). The differences are statistically significant. According to the SCURA (Fig 8), OM+ME had the best efficacy in endoscopic improvement, followed by OQC+QCE, QCE+OM.

#### TCM clinical syndrome integral

**Fig 9:**
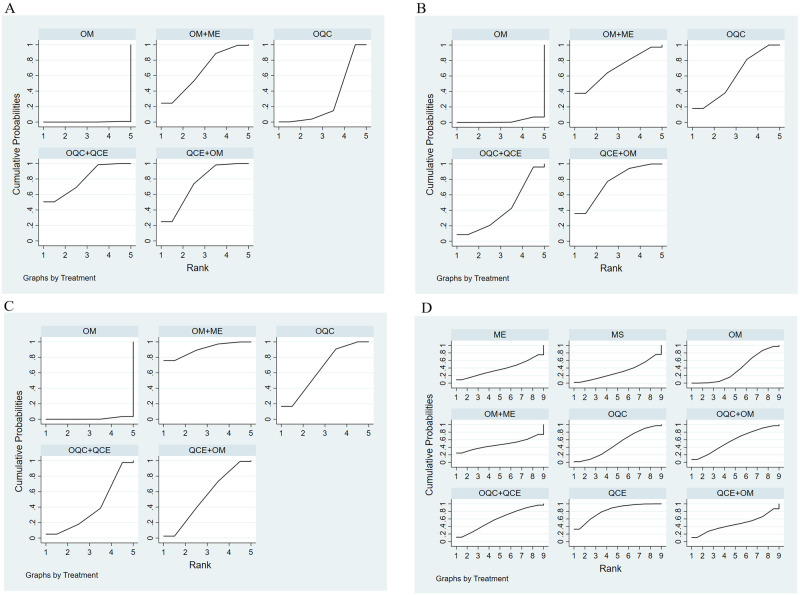
Surface under the cumulative raking curve of TCM syndrome and integral. (a) Abdominal pain; (b) bloody stool; (c) diarrhea; (d) adverse effects.

This NMA, which included 3 subgroups: abdominal pain, diarrhea, and bloody stool contained 7 trials with 5 therapies. For abdominal pain (Table 2), OQC+QCE is superior to OQC (SMD = 1.79, 95%CI = 1.03, 3.10) and OM (SMD = 2.85, 95%CI = 1.72, 4.70). QCE+OM (SMD = 2.64, 95%CI = 1.54, 4.70), OM+ME (SMD = 2.50, 95%CI = 1.20, 5.24) and OQC (SMD = 1.59, 95%CI = 1.26, 2.0) is better than OM. In terms of relieving diarrhea (Table 2), OM+ME (SMD = 2.81, 95%CI = 1.35, 5.82), OQC (SMD = 2.02, 95%CI = 1.60, 2.55) and QCE+OM (SMD = 1.90, 95%CI = 1.33, 3.19) had better efficacy than OM. As for the improvement of bloody stool (Table 2), QCE+OM (SMD = 2.09, 95%CI = 1.23, 3.52) and OQC (SMD = 1.78, 95%CI = 1.4, 2.24) was superior to OM. The SCURA plot revealed that OQC+QCE was the best therapy in relieving abdominal pain (Fig 9A), QCE+OM (Fig 9B) was the optimal intervention in avoiding bloody stool, and OM+ME showed the best efficacy in reducing diarrhea (Fig 9C).

#### Adverse effects

Totally, 19 trials with 9 treatments calculated the adverse effects. There were no significant statistical differences among all the therapies (Table 2). Based on the SCURA plot (Fig 9D), QCE was the most favorable intervention, OQC+QCE was second and OQC+OM was third.

### Quality evidences based on the GRADE system

The GRADE system with five elements was used to estimate the quality of evidence. Because of the unclear risk of bias and indirect comparison, the quality evaluation of clinical response was “low” (S3 Table).

## Discussion

Network meta-analysis can combine direct and indirect evidences to analyze multiple interventions and estimate the relative effects of all the included treatments from included trials when no head-to-head studies have been performed [51]. To our knowledge, this is the first NMA to evaluate the comparative efficacy and safety between CHM and Mesalazine in different administrations for the treatment of UC patients.

The NMA evidence revealed that OQC+OM was the best intervention in inducing clinical response; OQC+QCE had the best curative effect in the improvement of Mayo scores and the remission of abdominal pain; OM+ME was the optimal therapy in the endoscopic improvement and reducing diarrhea; QCE+OM was the best option in preventing bloody stool. In addition, QCE cause the less adverse effects and was the safest therapy among all the therapies. Therefore, QC could be considered as a complementary and alternative option in the management of UC, which provides more suggestions and guidance in the clinical decision.

The selection of medications is guided by disease severity and extension. Accordingly, a rapid step-up approach based on ulcerative colitis severity and treatment response is recommended. For patients with proctitis and left-sided colitis, suppositories and enema can work rapidly by targeting the site of inflammation in the splenic flexure and distal colon directly, while oral formulations or combined with an enema seems to have better therapeutic effects for patients with right-sided or extensive diseases [52–54]. This NMA indicated that the combination of topical and oral formulations was much better than either alone.

The exact pathogenesis of UC is still unclarified, with the colonocytes, mucous barrier and epithelial barrier defects, dysbiosis, a dysregulated immune response, and autoimmunity associated with cytotoxic autoantibodies possibly involved [55–59]. In TCM theory, the causes of UC can be concluded as two main aspects: external pathogen and internal deficiency. In active UC, external pathogens especially the dampness-heat invade the large intestine, damaging the intestinal mucosal and therefore leading to bloody stool. Besides, the invasion of dampness-heat will disturb the normal function of the large intestine and result in diarrhea. Therefore, the main principle of treatment in active UC should be “clear heat and eliminate dampness”, also known as “Qingre-Chushi” in TCM jargon.

The QC formulations mainly contain multiple herbs and the effective component is diverse. Modern pharmacological researches and experiments have indicated that QC formulations may potentially regulate the human body from various mechanisms and treat the disease. An animal experiment confirmed that the Feiyangchangweiyan capsule can modulate the OSM/OSMR pathway and regulate inflammatory factors to improve gut microbiota [60]. In acute/chronic UC models, Gegen Qinlian decoction can restore the colonic epithelium by maintaining mucosal homeostasis via bidirectional regulation of Notch signaling [61]. Evidence showed that Huanglian Jiedu decoction can suppress nuclear factor-κB signaling pathways, activate Nrf2 signaling pathways and enhance intestinal barrier function in acute UC mice [62]. Furthermore, clinical research also confirmed that QC can regulate the level of immune factors such as IL-17, IL-23, and the mark of inflammation such as TNF-α, CRP in UC patients, thus alleviating clinical symptoms [33, 36, 40].

Currently, several systematic reviews and meta-analyses were conducted to explore the efficacy of TCM in UC with the introduction and practice of evidence-based medicine [21]. However, these studies were varied in quality and were methodologically insufficient. In this NMA, the methodological quality of all RCTs was moderate and quality estimates based on the GRADE system showed “low”, which could originate from certain biases. Although all the patients were allocated randomly to interventions through a random number table or computer-generated sequences, only 4/21(19.05%) trials conducted allocation concealment, inevitably leading to selection bias. Besides, there were only 3/21(14.29%) trials using dummy placebo while 4/21(19.05%) trials were evaluated separately, so the participants and researcher would know the information of interventions. The lack of blind methods would result in performance bias and detection bias.

This study has some limitations. First, all the included studies were conducted in China, so it’s difficult to evaluate the efficacy of CHM in different races and regions. More large-scale, multicenter clinical trials should be conducted around the world in the future. Second, most of our included trials (16/21) were lack of long-term follow-up. Therefore, the efficacy and safety of CHM in long term use are still needed to be explored. Third, the formulations of CHM varied from each study, and the discrepancy may exist because of their source and preparation, which could influence the strength of evidence. Fourth, the criteria of clinical outcomes assessment such as clinical response and improvement of endoscopic were not consistent, which may cause uncertainty to the evidence.

## Conclusion

In summary, this study demonstrated that QC combined with mesalazine has a better effect than using mesalazine alone in inducing clinical response, improving Mayo scores, and alleviating clinical symptoms. In addition, oral formulation combined with topical is better than single administrations in alleviating symptoms and improving quality of life in UC patients. However, more high-quality, multicenter RCTs are necessary for the future to offer more powerful evidence.

## Supporting information

S1 Table PRISM checklist. (PDF)

S2 Table Composition of TCM decoction for included studies. (PDF)

S3 Table Grade of recommendations assessment, development and evaluation quality grading assessment. (PDF)

S1 File Search strategies of each database. (PDF)

S2 File The definition of clinical outcomes. (PDF)
