# Supplementary material for: Efficacy and safety of Qingre-Chushi therapies in active ulcerative colitis: A network meta-analysis
Source: PLoS One. 2021 Sep 20;16(9):e0257599. doi: 10.1371/journal.pone.0257599 (PMC8452044; doi:10.1371/journal.pone.0257599)
Supplement: S2 Table — (PDF) [file pone.0257599.s002.pdf]

Composition of TCM decoction for included studies

Study	Formulation	Source	Species, concentration	Quality control reported? (Y/N)	Chemical analysis reported? (Y/N)
Fan et.al 2020	Huofeng Qingchang decoction	Huangpu Affiliated Hospital of Guangdong Second Traditional Chinese Medicine Hospital	*Persicaria chinensis (L.) H.Gross*，30g<br>*Pteris multifida Poir.*，30g<br>*Scleromitrion diffusum (Willd.) R.J.Wang*，30g<br>*Patrinia scabiosifolia Link*，30g<br>Tuber of *Corydalis yanhusuo (Y.H.Chou & Chun C.Hsu) W.T.Wang ex Z.Y.Su & C.Y.Wu*，20g<br>*Ilex rotunda Thunb.*，20g<br>Root of *Saposhnikovia divaricata (Turcz. ex Ledeb.) Schischk.*，18g<br>Rhizome of *Atractylodes macrocephala*，15g<br>Root and Rhizome of *Glycyrrhiza uralensis Fisch. ex DC.*，6g	Y- Prepared according to Pharmacopoeia of the People's Republic of China	N
Shan 2020	Qingkui Yuyang decoction	Lianyungang Hospital of TCM	Root of *Pulsatilla chinensis (Bunge) Regel*，30g<br>*Ehizome* of *Coptis chinensis*，10g<br>Leaf of *Isatis tinctoria L.*，10g<br>Herb of *Euphorbia humifusa*，10g<br>Stem and branch of *Lonicera japonica Thunb.*，10g<br>Tuber of *Bletilla striata (Thunb.) Rchb.f.*，10g<br>Roots of *Sanguisorba officinalis L.*，10g<br>Branch and stem of *Senegalia catechu (L.f.) P.J.H.Hurter & Mabb*，10g<br>Root of *Panax notoginseng (Burkill) F.H.Chen*，10g<br>Resin of *Boswellia sacra Flück.*，10g	Y- Prepared according to Pharmacopoeia of the People's Republic of China	N
Wang 2020	QC formulation	Yunnan Province Hospital of TCM	Root of Astragalus mongholicus，30g<br>Root of Codonopsis pilosula，30g<br>Rhizome of *Conioselinum anthriscoides*，15g<br>Sterile fruit of *Gleditsia sinensis*，15g<br>Rhizome of *Atractylodes macrocephala*，20g<br>Rhizome of *Zingiber officinale*，15g<br>Rhizome of *Neopicrorhiza scrophulariiflora*，15g<br>*Erinaceus europaeus L*，10g<br>Herb of *Euphorbia humifusa*，30g<br>Rhizome of *Fagopyrum cymosum (Trevir.) Meisn.*，30g<br>Leaf of *Isatis tinctoria L.*，10g<br>Root and Rhizome of *Glycyrrhiza uralensis Fisch.*	Y- Prepared according to Pharmacopoeia of the People's Republic of China	N

			ex DC.，10g		
Xie et.al 2020	Oral formulation: Lichang decoction Enema formulation: Kuiyangling decoction	Shenzhen Traditional Chinese Medicine Hospital	Lichang decoction:<br>Root of Codonopsis pilosula，30g<br>Rhizome of *Atractylodes macrocephala*，15g<br>Cortex *Ailanthi.*，30g<br>Ripe kernel of *Coix lacryma-jobi var. ma-yuen (Rom.Caill.) Stapf*，30g<br>Rhizome and root of *Vincetoxicum mukdenense Kitag.*，30g<br>Ripe fruit of *Terminalia chebula Retz.*，15g<br>Root of *Panax notoginseng (Burkill) F.H.Chen*，10g<br>Tuber of *Bletilla striata (Thunb.) Rchb.f.* ，15g<br>Pollen of *Typha angustifolia L.*，15g<br>Flower bud of *Styphnolobium japonicum (L.) Schott*，15g<br>Root and Rhizome of *Glycyrrhiza uralensis Fisch. ex DC.*，10g<br>Kuiyang decoction:<br>Root of *Sophora flavescens Aiton*，30g<br>Root of *Panax notoginseng (Burkill) F.H.Chen*，10g<br>Tuber of *Bletilla striata (Thunb.) Rchb.f.* ，15g<br>Branch and stem of *Senegalia catechu (L.f.) P.J.H.Hurter & Mabb*，15g<br>Leaf of *Isatis tinctoria L.*，10g<br>Pollen of *Typha angustifolia L.*，15g<br>Flower *bud* of *Styphnolobium japonicum (L.) Schott*，15g<br>Cecidium of *Rhus chinensis Mill.*，10g<br>Alumen，10g	Y- Prepared according to Pharmacopoeia of the People's Republic of China	N
Zhong et.al 2020	Tiaochang Xiaoyan pill	Guangdong Province Traditional Chinese Medical Hospital	Root of Astragalus mongholicus<br>Rhizome of *Coptis chinensis*<br>*Scleromitrion diffusum (Willd.) R.J.Wang*<br>Root of *Aucklandia costus Falc.*<br>Root of *Lindera aggregata (Sims) Kosterm.*	Y- Prepared according to Pharmacopoeia of the People's Republic of China	N
Du 2019	Changyu enema recipe	Guangdong Efons Pharmaceutical co., Ltd	Tuber of *Bletilla striata (Thunb.) Rchb.f.* ，10g<br>Aerial parts of *Agrimonia pilosa Ledeb.*，6g<br>Bark of *Phellodendron amurense Rupr.*，6g<br>Root of *Sophora flavescens Aiton*，6g	Y- Prepared according to Pharmacopoeia of the People's Republic of	N

				China	
Ding 2019	Qingchang-Huashi decoction	Jiangsu Tianjiang Pharmaceutical co., Ltd	*Ehizome* of *Coptis chinensis*, 5g<br>Bark of *Magnolia* o*fficinalis Rehder & E.H.Wilson*, 6g<br>*Persicaria chinensis (L.) H.Gross*, 15g<br>Fruit of *Areca catechu L.*, 6g<br>*Scleromitrion diffusum (Willd.) R.J.Wang*, 15g<br>Root of *Auckl*and*ia costus Falc.*, 6g<br>Rhizome of *Atractylodes macrocephala*, 10g<br>Root of *Paeonia lactiflora Pall.*, 15g<br>Root of *Bupleurum chinense DC.*, 6g<br>Root of *Angelica sinensis (Oliv.) Diels*, 10g<br>*Plantago asiatica L.*, 6g<br>Root and Rhizome of *Glycyrrhiza uralensis Fisch. ex DC.*, 6g	Y- Prepared according to Pharmacopoeia of the People's Republic of China	N
Jia 2019	Qingxiao decoction	Inner Mongolia People's Hospital	Root of *Scutellaria baicalensis Georgi*, 20g<br>Bark of *Phellodendron amurense Rupr.*, 20g<br>Stem and branch of *Lonicera japonica Thunb.*, 20g<br>Fruit of *Forsythia suspensa (Thunb.) Vahl*, 20g<br>*Taraxacum mongolicum H*and. *-Mazz.*, 20g<br>Root*s* of *Sanguisorba* o*fficinalis L.*, 30g<br>Fruit of *Prunus mume (Siebold) Siebold & Zucc.*, 20g<br>Rhizome and root of *Rheum palmatum L.*, 20g<br>Leaf of *Isatis tinctoria L.*, 30g<br>Leaf of *Platycladus orientalis (L.) Franco*, 20g<br>Aerial parts of *Agrimonia pilosa Ledeb.*, 30g<br>Root of *Sophora flavescens Aiton*, 30g<br>*Alumen*, 5g	Y- Prepared according to Pharmacopoeia of the People's Republic of China	N
Wang 2019	Jianpi-Qingchang decoction	Mianyang Hospital of TCM	Root of *Pulsatilla chinensis (Bunge) Regel*, 20g<br>Root*s* of *Sanguisorba* o*fficinalis L.*, 30g<br>Root of *Paeonia lactiflora Pall.*, 25g<br>Rhizome of *Atractylodes lancea (Thunb.) DC.*, 12g<br>*Pericarp* of *Citrus reticulata Blanco*, 10g<br>Fruit of *Citrus × aurantium L.*, 20g<br>Root of Codonopsis pilosula, 20g<br>Rhizome of *Smilax glabra Roxb.*, 15g<br>Rhizome of *Zingiber* o*fficinale*, 6g<br>Rhizome of *Atractylodes macrocephala*, 15g<br>Root of *Scutellaria baicalensis Georgi*, 15g<br>Root and Rhizome of *Glycyrrhiza uralensis Fisch. ex DC.*, 5g	Y- Prepared according to Pharmacopoeia of the People's Republic of China	N

Wu 2019	QC formulation	Affiliated Hospital of Jiangxi University of TCM	Leaf of *Isatis tinctoria L.*, 30g<br>*Concha Ostreae*, 60g<br>Ripe seed of *Celosia argentea L.*, 60g<br>Branch and stem of *Senegalia catechu (L.f.) P.J.H.Hurter & Mabb*, 60g<br>Bark of *Phellodendron amurense Rupr.*, 30g<br>*Patrinia scabiosifolia Link*, 30g	Y- Prepared according to Pharmacopoeia of the People's Republic of China	N
Zhang 2019	Pulsatilla decoction	Guangdong Efons Pharmaceutical co., Ltd	Root of *Pulsatilla chinensis (Bunge) Regel*, 10g<br>Bark of *Phellodendron amurense Rupr.*, 3g<br>*Ehizome* of *Coptis chinensis*, 6g<br>*Branch* of *stem* of *Fraxinus chinensis Roxb.*, 10g	Y- Prepared according to Pharmacopoeia of the People's Republic of China	N
Feng et.al 2018	Qushi-Qingchang recipe<br>Qingre-Lishi enema recipe	Yueyang Hospital of Integrated Traditional Chinese and Western Medicine	Qushi-Qingchang recipe:<br>Root of *Pueraria montana var. lobata (Willd.) Maesen & S.M.Almeida ex Sanjappa & Predeep*, 30g<br>Aerial parts of *Portulaca oleracea L.*, 30g<br>Bark of *Phellodendron amurense Rupr.*, 9g<br>Root of *Scutellaria baicalensis Georgi*, 9g<br>*Ehizome* of *Coptis chinensis*, 9g<br>Root and Rhizome of *Glycyrrhiza uralensis Fisch. ex DC.*, 6g<br>Qingre-Lishi enema recipe:<br>Root and Rhizome of *Reynoutria japonica Houtt.*, 20g<br>*Taraxacum mongolicum H*and. *-Mazz.*, 20g<br>Root of *Sophora flavescens Aiton*, 10g<br>Bark of *Phellodendron amurense Rupr.*, 10g	Y- Prepared according to Pharmacopoeia of the People's Republic of China	N
Yao 2018	Changyu enema recipe	Guangdong Efons Pharmaceutical co., Ltd	Tuber of *Bletilla striata (Thunb.) Rchb.f.*, 10g<br>Aerial parts of *Agrimonia pilosa Ledeb.*, 6g<br>Bark of *Phellodendron amurense Rupr.*, 6g<br>Root of *Sophora flavescens Aiton*, 6g	Y- Prepared according to Pharmacopoeia of the People's Republic of China	N
Zhang et.al 2018	Qinghuachang recipe and No.3 enema recipe	The Second Affiliated Hospital of Fujian University of TCM	Aerial parts of *Agrimonia pilosa Ledeb.*<br>*Ehizome* of *Coptis chinensis*<br>Roots of *Sanguisorba officinalis L.*<br>Root of *Paeonia anomala subsp. veitchii (Lynch) D.Y.Hong & K.Y.Pan*<br>Fruit of *Wurfbainia vera (Blackw.) Skornick. & A.D.Poulsen*	Y- Prepared according to Pharmacopoeia of the People's Republic of China	N

			Bark of *Magnolia officinalis Rehder & E.H.Wilson*<br>Aerial parts of *Artemisia capillaris Thunb.*<br>Aerial parts of *Eupatorium fortunei Turcz.*<br>Ripe kernel of *Coix lacryma-jobi var. ma-yuen (Rom.Caill.) Stapf*<br>Seed of *Lablab purpureus subsp. purpureus*<br>Rhizome of *Smilax glabra Roxb.*<br>*Pericarp* of *Citrus reticulata Blanco*<br>Branch and stem of *Senegalia catechu (L.f.) P.J.H.Hurter & Mabb*<br>Root and Rhizome of *Glycyrrhiza uralensis Fisch. ex DC.*<br>*Crystal produced from the branches* and *leaves* of *Cinnamomum camphora (L.) J.Presl*		
Dai et.al 2017	Jianpi-Qingchang decoction	Affiliated Long Hua Hospital of Shanghai University of Traditional Chinese Medicine	Root of Astragalus mongholicus，30g<br>*Ehizome* of *Coptis chinensis*，3g<br>Root of Codonopsis pilosula，15g<br>Aerial parts of *Portulaca oleracea L.*，30g<br>Root*s* of *Sanguisorba officinalis L.*，15g<br>Root of *Panax notoginseng (Burkill) F.H.Chen*，6g<br>Tuber of *Bletilla striata (Thunb.) Rchb.f.* ，3g<br>Root of *Auckl*and*ia costus Falc.*，6g<br>Root and Rhizome of *Glycyrrhiza uralensis Fisch. ex DC.*，6g	Y- Prepared according to Pharmacopoeia of the People's Republic of China	N
Qin 2017	Qingre-Chushi decoction	Affiliated Dongzhimen Hospital of Beijing University of Chinese Medicine	Root of *Saposhnikovia divaricata (Turcz. ex Ledeb.) Schischk.*，10g<br>*Aerial part* of *Nepeta tenuifolia Benth.* ，10g<br>Root of *Pulsatilla chinensis (Bunge) Regel*，10g<br>Aerial parts of *Portulaca oleracea L.*，20g<br>*Patrinia scabiosifolia Link*，20g<br>Rhizome of *Atractylodes macrocephala*，15g<br>Root of *Paeonia lactiflora Pall.*，12g<br>Root and Rhizome of *Glycyrrhiza uralensis Fisch. ex DC.*，10g	Y- Prepared according to Pharmacopoeia of the People's Republic of China	N
Bao et.al 2015	Gegen-Qinlian decoction	Gansu Province Hospital of TCM	Root of Codonopsis pilosula，20g<br>Rhizome of *Atractylodes macrocephala*，20g<br>Rhizome of *Zingiber officinale*，15g<br>*Ehizome* of *Coptis chinensis*，5g<br>*Halloysitum Rubrum*，20g<br>Root of *Paeonia lactiflora Pall.*，20g<br>Ripe kernel of *Coix lacryma-jobi var. ma-yuen (Rom.Caill.) Stapf*，30g<br>Aerial parts of *Portulaca oleracea L.*，20g<br>Rhizome of *Smilax glabra Roxb.*，20g	Y- Prepared according to Pharmacopoeia of the People's Republic of China	N

			Pericarp of *Citrus reticulata Blanco*，10g<br>Root of *Aucklandia costus Falc.*，10g<br>Root and Rhizome of *Glycyrrhiza uralensis Fisch. ex DC.*，5g		
Yang 2014	Jiaodai decoction	Affiliated Shandong Province Qianfoshan Hospital of Shandong University	Leaf of *Isatis tinctoria L.*，31g<br>Bark of *Phellodendron amurense Rupr.*，31g<br>Root and Rhizome of *Glycyrrhiza uralensis Fisch. ex DC.*，31g<br>Gypsum Fibrosum，31g<br>*Aerial part* of *Mentha canadensis L.*，1.5g	Y- Prepared according to Pharmacopoeia of the People's Republic of China	N
Gong et.al 2012	Fufang-Kushen colon-coated capsule	Beijing Zhonghui Pharmaceutical co., Ltd	Root of *Sophora flavescens Aiton*<br>Roots of *Sanguisorba officinalis L.*<br>Leaf of *Isatis tinctoria L.*<br>Tuber of *Bletilla striata (Thunb.) Rchb.f.*<br>Root and Rhizome of *Glycyrrhiza uralensis Fisch. ex DC.*	Y- Prepared according to Pharmacopoeia of the People's Republic of China	N
Liu 2011	Shaoyaoqiwu recipe	Hunan Spring 9 Department of modern traditional Chinese Medicine Co., Ltd.	Root of *Paeonia lactiflora Pall.*，10g<br>Root of *Angelica sinensis (Oliv.) Diels*，10g<br>Root of *Scutellaria baicalensis Georgi*，9g<br>Ehizome of *Coptis chinensis*，9g<br>Bark of *Phellodendron amurense Rupr.*，6g<br>Fruit of *Areca catechu L.*，10g<br>Root of *Aucklandia costus Falc.*，5g<br>Root and Rhizome of *Glycyrrhiza uralensis Fisch. ex DC.*，6g	Y- Prepared according to Pharmacopoeia of the People's Republic of China	N
Tong et.al 2011	Fufang-Kushen colon-coated capsule	Beijing Zhonghui Pharmaceutical co., Ltd	Root of *Sophora flavescens Aiton*<br>Roots of *Sanguisorba officinalis L.*<br>Leaf of *Isatis tinctoria L.*<br>Tuber of *Bletilla striata (Thunb.) Rchb.f.*<br>Root and Rhizome of *Glycyrrhiza uralensis Fisch. ex DC.*	Y- Prepared according to Pharmacopoeia of the People's Republic of China	N

The frequency of included herbs

**Chinese herbs**	**frequency**
Root and Rhizome of Glycyrrhiza uralensis Fisch. ex DC	15
Bark of Phellodendron amurense Rupr	9
Leaf of Isatis tinctoria L	9
Tuber of Bletilla striata (Thunb.) Rchb.f	9
Ehizome of Coptis chinensis	8
Roots of Sanguisorba officinalis L	8
Rhizome of Atractylodes macrocephala	7

cRoot of Sophora flavescens Aiton	7
Root of Aucklandia costus Falc	5
Root of Codonopsis pilosula	5
Root of Paeonia lactiflora Pall	5
Aerial parts of Agrimonia pilosa Ledeb	4
Aerial parts of Portulaca oleracea L	4
Branch and stem of Senegalia catechu (L.f.) P.J.H.Hurter & Mabb	4
Root of Panax notoginseng (Burkill) F.H.Chen	4
Root of Pulsatilla chinensis (Bunge) Regel	4
Root of Scutellaria baicalensis Georgi	4
Patrinia scabiosifolia Link	3
Pericarp of Citrus reticulata Blanco	3
Rhizome of Smilax glabra Roxb	3
Rhizome of Zingiber officinale	3
Ripe kernel of Coix lacryma-jobi var. ma-yuen (Rom.Caill.) Stapf	3
Root of Astragalus mongholicus	3
Scleromitrion diffusum (Willd.) R.J.Wang	3
Alumen	2
Bark of Magnolia officinalis Rehder & E.H.Wilson	2
Flower bud of Styphnolobium japonicum (L.) Schott	2
Fruit of Areca catechu L	2
Herb of Euphorbia humifusa	2
Persicaria chinensis (L.) H.Gross	2
Pollen of Typha angustifolia L	2
Root of Angelica sinensis (Oliv.) Diels	2
Root of Saposhnikovia divaricata (Turcz. ex Ledeb.) Schischk	2
Stem and branch of Lonicera japonica Thunb	2
Taraxacum mongolicum Hand. -Mazz	2
Aerial part of Mentha canadensis L	1
Aerial part of Nepeta tenuifolia Benth	1
Aerial parts of Artemisia capillaris Thunb.	1
Aerial parts of Eupatorium fortunei Turcz.	1
Branch of stem of Fraxinus chinensis Roxb	1
Cecidium of Rhus chinensis Mill	1
Concha Ostreae	1
Cortex Ailanthi	1
Crystal produced from the branches and leaves of Cinnamomum camphora (L.) J.Presl	1
Erinaceus europaeus L	1
Fruit of Citrus × aurantium L	1
Fruit of Forsythia suspensa (Thunb.) Vahl	1
Fruit of Prunus mume (Siebold) Siebold & Zucc	1
Fruit of Wurfbainia vera (Blackw.) Skornick. & A.D.Poulsen	1
Gypsum Fibrosum	1

Halloysitum Rubrum	1
Ilex rotunda Thunb	1
Kuiyang decoction:	1
Leaf of Platycladus orientalis (L.) Franco	1
Plantago asiatica L	1
Pteris multifida Poir	1
Qingre-Lishi enema recipe:	1
Resin of Boswellia sacra Flück	1
Rhizome and root of Rheum palmatum L	1
Rhizome and root of Vincetoxicum mukdenense Kitag	1
Rhizome of Atractylodes lancea (Thunb.) DC	1
Rhizome of Conioselinum anthriscoides	1
Rhizome of Coptis chinensis	1
Rhizome of Fagopyrum cymosum (Trevir.) Meisn	1
Rhizome of Neopicrorhiza scrophulariiflora	1
Ripe fruit of Terminalia chebula Retz	1
Ripe seed of Celosia argentea L	1
Root and Rhizome of Reynoutria japonica Houtt	1
Root of Bupleurum chinense DC	1
Root of Lindera aggregata (Sims) Kosterm.	1
Root of Paeonia anomala subsp. veitchii (Lynch) D.Y.Hong & K.Y.Pan	1
Root of Pueraria montana var. lobata (Willd.) Maesen & S.M.Almeida ex Sanjappa & Predeep	1
Seed of Lablab purpureus subsp. purpureus	1
Sterile fruit of Gleditsia sinensis	1
Tuber of Corydalis yanhusuo (Y.H.Chou & Chun C.Hsu) W.T.Wang ex Z.Y.Su & C.Y.Wu	1
