# Supplementary material for: Efficacy and safety of Qingre-Chushi therapies in active ulcerative colitis: A network meta-analysis
Source: PLoS One. 2021 Sep 20;16(9):e0257599. doi: 10.1371/journal.pone.0257599 (PMC8452044; doi:10.1371/journal.pone.0257599)
Supplement: S3 Table — (PDF) [file pone.0257599.s003.pdf]

QC compared to Mesalazine for Ulcerative colitis(UC)

Patient or population: patients with Ulcerative colitis(UC)
Settings:
Intervention: QC
Comparison: Mesalazine

Outcomes	Illustrative comparative risks* (95% CI) Assumed risk **Mesalazine**	Corresponding risk **QC**	Relative effect (95% CI)	No of Participants (studies)	Quality of the evidence (GRADE)	Comments
**clinical response**	**Study population**		**RR 1.15** (1.09 to 1.21)	1383 (16 studies)	⊕⊕⊖⊖ **low**[1,2,3]	
	**784 per 1000**	**902 per 1000** (855 to 949)				
	**Moderate**					
	**833 per 1000**	**958 per 1000** (908 to 1000)				
**clinical response -OM VS OQC**	**Study population**		**RR 1.17** (1.08 to 1.26)	829 (8)		
	**740 per 1000**	**866 per 1000** (799 to 932)				
	**Moderate**					
	**793 per 1000**	**928 per 1000** (856 to 999)				
**clinical response- OM VS OQC+OM**	**Study population**		**RR 1.26** (0.93 to 1.69)	27 (1)		
	**786 per 1000**	**990 per 1000** (731 to 1000)				
	**Moderate**					
	**786 per 1000**	**990 per 1000** (731 to 1000)				
**Clinical response- OM VS OQC+QCE**	**Study population**		**RR 1.26** (0.99 to 1.61)	65 (1)		
	**719 per 1000**	**906 per 1000** (712 to 1000)				
	**Moderate**					
	**719 per 1000**	**906 per 1000** (712 to 1000)				
**Clinical response- OM VS QCE**	**Study population**		**RR 1.12** (0.93 to 1.34)	64 (1)		
	**938 per 1000**	**1000 per 1000** (872 to 1000)				
	**Moderate**					
	**938 per 1000**	**1000 per 1000** (872 to 1000)				
**Clinical response- OM VS QCE+OM**	**Study population**		**RR 1.12** (0.93 to 1.34)	62 (1)		
	**839 per 1000**	**939 per 1000** (780 to 1000)				
	**Moderate**					
	**839 per 1000**	**940 per 1000** (780 to 1000)				
**Clinical response- ME VS QCE**	**Study population**		**RR 1.12** (0.93 to 1.34)	62 (1)		
	**839 per 1000**	**939 per 1000** (780 to 1000)				
	**Moderate**					
	**839 per 1000**	**940 per 1000** (780 to 1000)				
**Clinical response- MS VS QCE**	**Study population**		**RR 1.04** (0.94 to 1.15)	182 (3)		
	**868 per 1000**	**903 per 1000** (816 to 998)				
	**Moderate**					
	**839 per 1000**	**873 per 1000** (789 to 965)				
**Clinical response- OM+ME VS OM+QCE**	**Study population**		**RR 1.16** (0.98 to 1.38)	60 (1)		
	**833 per 1000**	**967 per 1000** (817 to 1000)				
	**Moderate**					
	**833 per 1000**	**966 per 1000** (816 to 1000)				
**Clinical response- OQC VS OQC+OM**	**Study population**		**RR 1.47** (1.04 to 2.06)	31 (1)		
	**667 per 1000**	**980 per 1000** (693 to 1000)				
	**Moderate**					
	**667 per 1000**	**980 per 1000** (694 to 1000)				

*The basis for the **assumed risk** (e.g. the median control group risk across studies) is provided in footnotes. The **corresponding risk** (and its 95% confidence interval) is based on the assumed risk in the comparison group and the **relative effect** of the intervention (and its 95% CI).

CI: Confidence interval; **RR:** Risk ratio;

GRADE Working Group grades of evidence
High quality: Further research is very unlikely to change our confidence in the estimate of effect.
Moderate quality: Further research is likely to have an important impact on our confidence in the estimate of effect and may change the estimate.
Low quality: Further research is very likely to have an important impact on our confidence in the estimate of effect and is likely to change the estimate.
Very low quality: We are very uncertain about the estimate.

[1] Blinding with less literature
[2] Indirect comparsion
[3] Different interventions
