# Supplementary material for: Efficacy and safety of Qingre-Chushi therapies in active ulcerative colitis: A network meta-analysis
Source: PLoS One. 2021 Sep 20;16(9):e0257599. doi: 10.1371/journal.pone.0257599 (PMC8452044; doi:10.1371/journal.pone.0257599)
Supplement: S1 File — (PDF) [file pone.0257599.s004.pdf]

Search strategies of each database

PubMed:

#1 ulcerative colitis [SH]
#2 UC [SH]
#3 inflammatory bowel disease [SH]
#4 IBD [SH]
#5 exp proctitis [SH]
#6 exp sigmoiditis
#7 exp pancolitis [SH]
#8 #1 or #2 or #3 or #4 or #5 or #6 or #7
#9 traditional Chinese medicine [SH]
#10 exp Chinese medicinal herb [SH]
#11 exp traditional Chinese herbal formula [SH]
#12 exp herbs [SH]
#13 #9 or #10 or #11 or #12
#14 randomized controlled trial [PT]
#15 controlled clinical trial [PT]
#16 randomized [TIAB]
#17 randomly [TIAB]
#18 trial [TIAB]
#19 #14 or #15 or #16 or #17 or #18
#20 #8 and #13 and #19

Annotation: Mesh Subheadings [SH]; Publication Type [PT]; Title/Abstract [TIAB]

Springer Link:

#1 traditional Chinese medicine OR Chinese medicinal herb OR traditional Chinese herbal formula OR herbs

#2 ulcerative colitis OR UC OR inflammatory bowel disease OR IBD

#3 randomized controlled trial OR controlled clinical trial OR randomized

#4 #1 AND #2 AND #3

EMBASE:

#1 (irritable bowel syndrome or IBS or irritable bowel disease or irritable colitis). ab.

#2 exp functional bowel disease/ or exp allergic colitis/ or exp colon allergy/

#3 1 or 2

#4 (traditional Chinese medicine). ab.

#5 exp Chinese medicinal herb/ or exp traditional Chinese herbal formula/ or exp herbs/

#6 4 or 5

#7 (random* OR factorial* OR crossover*). ab.

#8 exp double-blind procedure/ or exp randomized controlled trial/ or single-blind procedure/

#9 7 or 8

10 3 and 6 and 9

Cochrane library:

#1 ulcerative colitis OR UC

#2 traditional Chinese medicine

#3 randomized controlled trial OR controlled clinical trial OR randomized

#4 #1 AND #2 AND #3

CNKI (China National Knowledge Infrastructure):

#1 ulcerative colitis OR UC

#2 traditional Chinese medicine

#3 randomized controlled trial OR controlled clinical trial OR randomized

#4 #1 AND #2 AND #3

CBM (Chinese Biomedicine Database)

#1 ulcerative colitis OR UC

#2 traditional Chinese medicine

#3 randomized controlled trial OR controlled clinical trial OR randomized

#4 #1 AND #2 AND #3

Wanfang:

#1 ulcerative colitis OR UC

#2 traditional Chinese medicine

#3 randomized controlled trial OR controlled clinical trial OR randomized

#4 #1 AND #2 AND #3
