# Supplementary material for: Efficacy and safety of Qingre-Chushi therapies in active ulcerative colitis: A network meta-analysis
Source: PLoS One. 2021 Sep 20;16(9):e0257599. doi: 10.1371/journal.pone.0257599 (PMC8452044; doi:10.1371/journal.pone.0257599)
Supplement: S2 File — (PDF) [file pone.0257599.s005.pdf]

The definition of clinical outcomes

Study ID	Clinical response	Mayo scores	Endoscopic improvement	TCM syndrome integral	Adverse effects
**Fan et.al 2020**	MS≤30% from baseline or IMS≥3; IRB≥1 or RB=0/1	N/A	MES	Reported	N/A
**Shan 2020**	MS≤30% from baseline or IMS≥3; IRB≥1 or RB=0/1	Reported	MES	Reported	Reported
**Wang 2020**	MS≤30% from baseline or IMS≥3; IRB≥1 or RB=0/1	Reported	MES	Reported	Reported
**Xie et.al 2020**	N/A	Reported	MES	N/A	Reported
**Zhong et.al 2020**	MS≤30% from baseline or IMS≥3; IRB≥1 or RB=0/1	Reported	N/A	N/A	Reported
**Ding 2019**	N/A	Reported	BS	Reported	Reported
**Du 2019**	MS≤30% from baseline	Reported	BS	N/A	Reported
**Jia 2019**	MS≤30% from baseline	N/A	N/A	N/A	Reported
**Wang 2019**	MS≤30% from baseline or IMS≥3; IRB≥1 or RB=0/1	Reported	BS	Reported	Reported
**Wu 2019**	MS≤30% from baseline or IMS≥3; IRB≥1 or RB=0/1	Reported	MES	Reported	Reported
**Zhang 2019**	MS≤30% from baseline	Reported	BS	N/A	Reported
**Feng 2018**	N/A	Reported	N/A	Reported	Reported
**Yao 2018**	BS≤30% from baseline	N/A	N/A	Reported	N/A
**Zhang et.al 2018**	MS≤30% from baseline or IMS≥3; IRB≥1 or RB=0/1	Reported	N/A	N/A	Reported
**Dai et.al 2017**	N/A	Reported	N/A	N/A	Reported
**Qin 2017**	N/A	Reported	BS	Reported	Reported
**Bao et.al 2015**	TCM syndrome	N/A	N/A	Reported	Reported

	integral ≤30% from baseline				
**Yang 2014**	IBS≥1	N/A	BS	N/A	Reported
**Gong 2012**	MS≤30% from baseline or IMS≥3; IRB≥1 or RB=0/1	Reported	N/A	N/A	Reported
**Liu 2011**	Mild symptoms, mild inflammatory of mucosal	N/A	N/A	N/A	Reported
**Tong et.al 2011**	Mild symptoms, mild inflammatory of mucosal	Reported	N/A	N/A	Reported

MS: Mayo scores; IMS: improvement of Mayo scores; MES; Mayo endoscopic scores; RB: rectal bleeding; IRB: improvement of rectal bleeding; BS: Baron scores; TCM: traditional Chinese medicine

1.**Mayo scores**

Mayo scoring system for assessment of ulcerative colitis activity. *

Stool frequency†

0 = Normal no. of stools for this patient

1 = 1–2 stools more than normal

2 = 3–4 stools more than normal

3 = 5 or more stools more than normal

Subscore, 0–3

Rectal bleeding‡

0 =No blood seen

1 = Streaks of blood with stool less than half the time

2 = Obvious blood with stool most of the time

3 = Blood alone passes

Subscore, 0–3

Findings on endoscopy

0 = Normal or inactive disease

1 =Mild disease (erythema, decreased vascular pattern, mild friability)

2 =Moderate disease (marked erythema, lack of vascular pattern, friability, erosions)

3 = Severe disease (spontaneous bleeding, ulceration)

Subscore, 0–3

Physician's global assessment§

0 = Normal

1 =Mild disease

2 =Moderate disease

3 = Severe disease

Subscore, 0–3

* The Mayo score ranges from 0 to 12, with higher scores indicating more severe disease.

2.Baron scores

0 = Normal

1 = mild disease (no bleeding either spontaneously or to light touch)

2 = moderate disease (bleeding to light touch, but no spontaneous bleeding seen ahead of instrument at initial inspection)

3 = Severe disease (spontaneous bleeding seen ahead of instrument at initial inspection with bleeding to light tough)

3.TCM syndrome integral

Main symptoms	Normal (0)	Mild (1)	Moderate (2)	Severity (3)
**Diarrhea**	No	< 3 per day	3-6 per day	> 6 per day
**Abdominal pain**	No	Slightly dull pain, occasionally	Dull or swelling pain, couple times per day	Sharp pain, repeated
**Bloody stool**	No	Streaks of bloody stool	Obvious of bloody stool	All of bloody stool or with fresh blood
